# Evaluation of the beneficial effects of a GABA-based product containing *Melissa officinalis* on post-inflammatory irritable bowel syndrome: a preclinical study

**DOI:** 10.3389/fphar.2024.1466824

**Published:** 2024-09-20

**Authors:** Elena Lucarini, Laura Benvenuti, Clelia Di Salvo, Vanessa D’Antongiovanni, Carolina Pellegrini, Giulia Valdiserra, Clara Ciampi, Luca Antonioli, Christian Lambiase, Lorenzo Cancelli, Antonio Grosso, Lorenzo Di Cesare Mannelli, Massimo Bellini, Carla Ghelardini, Matteo Fornai

**Affiliations:** ^1^ Pharmacology and Toxicology Section, Department of Neuroscience, Psychology, Drug Research and Child Health, Neurofarba, University of Florence, Florence, Italy; ^2^ Department of Clinical and Experimental Medicine, University of Pisa, Pisa, Italy; ^3^ Department of Translational Research, New Technologies in Medicine and Surgery, University of Pisa, Pisa, Italy

**Keywords:** irritable bowel syndrome, colitis, GABA, *Melissa officinalis*, abdominal pain

## Abstract

**Introduction:**

Visceral pain represents the most common digestive issue, frequently resulting from long-term inflammation, such as inflammatory bowel diseases. The lack of effective drugs prompted search of new therapeutic approaches. In this regard, gamma-aminobutyric acid (GABA) and *Melissa officinalis* (Mo) appear as excellent candidates as they were recognized to have several positive effects on the digestive system. The aim of this research was to evaluate the effects of a compound containing GABA and Mo (GABA-Mo 5:1) in inflammation-induced intestinal damage and visceral pain.

**Methods:**

Colitis was induced in rats by intrarectal 2,4-dinitrobenzenesulfonic acid (DNBS) administration. DNBS-treated animals received GABA-Mo (80 mg/kg BID), starting 3 days before DNBS administration, until 14 days after colitis induction (preventive protocol), or starting 7 days after DNBS until day 21 (curative protocol). Visceral pain was assessed by measuring the viscero-motor response (VMR) and the abdominal withdrawal reflex (AWR) to colorectal distension on day 7, 14 (both protocols) and 21 (curative protocol) after DNBS administration.

**Results:**

In the preventive protocol, GABA-Mo reduced AWR at day 14 but had no effect on VMR. In the spinal cord, treatment with GABA-Mo significantly prevented microglia reactivity (Iba-1 positive cells). In the colon, the supplement significantly decreased malondialdehyde (MDA, index of oxidative stress) and IL-1β levels and counteracted the decreased expression of claudin-1. Moreover, GABA-Mo normalized the increased levels of plasma lipopolysaccharide binding protein (LBP, index of altered intestinal permeability). In the curative protocol, GABA-Mo significantly counteracted visceral hypersensitivity persistence in DNBS-treated animals (day 14 and 21). In the spinal cord, GABA-Mo significantly reduced GFAP positive cell density (astrocytes). Histological evaluations highlighted a mild but significant effect of GABA-Mo in promoting healing from DNBS-induced colon damage. Colonic MDA and myeloperoxidase (index of leukocyte infiltration) levels were reduced, while the decreased colonic claudin-1 expression was normalized. In addition, the increased levels of plasma LBP were normalized by GABA-Mo administration.

**Discussion:**

In conclusion GABA-Mo, particularly in the curative protocol, was able to reduce visceral pain and intestinal inflammation, likely through a reinforcement of intestinal barrier integrity, thus representing a suitable approach for the management of abdominal pain, especially in the remission stages of colitis.

## 1 Introduction

Chronic abdominal pain can result from gastrointestinal infections or prolonged inflammatory processes, such as in inflammatory bowel diseases (IBDs), but in many patients, negative diagnostic test results lead to the diagnosis of irritable bowel syndrome (IBS) (Giamberardino et al., 2010; Spiller and Major, 2016). The establishment of abdominal pain is due to changes that begin with hypersensitivity of the primary sensory neurons, which innervate the gastrointestinal tract, and afterwards reach the central nervous system (CNS) (Srinath et al., 2012). Inflammation does not completely explain altered perception of pain in patients affect by IBD, as 20%–50% of patients complain about abdominal pain in the clinical remission stage (Minderhoud et al., 2004; Srinath et al., 2012). The poor correlations between reported abdominal pain intensity and IBD activity indices reinforce the complex nature of this type of pain and connect it to diarrheal-predominant IBS (IBS-D) (Farrokhyar et al., 2006; Spiller and Major, 2016). IBS is a functional bowel disorder characterized by the presence of chronic/recurrent abdominal pain or discomfort, with altered bowel habits and consequent anomalies in stool frequency and form (Portincasa et al., 2019). Pathogenesis of IBS is only partially understood, even though there is some evidence that changes in the digestive motility and secretion, visceral hypersensitivity, abnormalities of enteroendocrine and immune systems, genetic factors, infections, alterations of the intestinal microbiota and immune response derangement could play a role in this functional disease (Bellini M et al., 2014; Aguilera-Lizarraga et al., 2022). Although IBD and IBS-D are different pathologies, there is a clinical overlap between these, therefore, the therapeutic approach to relieve pain in IBD and IBS is often the same (Dockerty et al., 2011; Spiller and Major, 2016). Unfortunately, current therapies offer little benefit for abdominal pain, in addition to the undesired and serious side effects they can produce in patients (Catanzaro et al., 2015; Bellini et al., 2020; Camilleri and boeckxstaens, 2023). As the pain management remains unsatisfactory, there is a growing demand for the development of effective treatments. Considering that post-inflammatory visceral hypersensitivity is sustained by alterations in the enteric barrier and immune response (Carabotti et al., 2015; Cervero, 2014), a product able to protect the intestinal mucosa by indirectly controlling inflammation and at the same time modulating immune response could be the answer. In this regard, gamma-aminobutyric acid (GABA) is known to exert a number of beneficial effects at digestive level. For instance, GABA has a protective role during inflammatory processes by modulating cytokine production (Gros et al., 2021). Moreover, products containing GABA have been proven to strengthen IEB and to modulate immune-inflammatory response (Sokovic Bajic et al., 2019; Cataldo et al., 2020). Based on the above considerations, the aim of the present study was to evaluate the protective effect of GABA-containing preparation on the intestinal inflammation, mucosal barrier impairment and visceral pain development in the rat pre-clinical model of colitis induced by DNBS. The presence of *Melissa officinalis* in the preparation is expected to support the beneficial effects of GABA, either by preventing its metabolism *in vivo* (Awad et al., 2007) or by a direct modulation of inflammation and nociception (Guginski et al., 2009; Dolatabadi et al., 2018; Zam et al., 2022; Chindo et al., 2024).

## 2 Materials and methods

### 2.1 Animals

For all the experiments described below, male Sprague–Dawley rats (Envigo, Varese, Italy), weighing approximately 220–250 g at the beginning of the experimental procedure, were used. Animals were housed in CeSAL (Centro Stabulazione Animali da Laboratorio, University of Florence, Florence, Italy) and used at least 1 week after their arrival. Four rats were housed per cage (size 26 × 41 cm); animals were fed a standard laboratory diet and tap water *ad libitum*, and kept at 23°C ± 1°C with a 12 h light/dark cycle, light at 7 a.m. All animal manipulations were carried out according to the Directive 2010/63/EU of the European Parliament and the European Union Council (22 September 2010) on the protection of animals employed for scientific purposes. The ethical policy of the University of Florence complies with the Guide for the Care and Use of Laboratory Animals of the US National Institutes of Health (NIH Publication number 85–23, revised 1996, University of Florence assurance number: A5278-01). Formal approval to conduct the described experiments was obtained from the Animal Subjects Review Board of the University of Florence (543/2017-PR). Experiments involving animals have been reported according to ARRIVE guidelines (McGrath and Lilley, 2015). All efforts were made to minimize animal suffering and to reduce the number of employed animals.

### 2.2 Induction of experimental colitis

Colitis has been induced in rats in conformity with the method described by Fornai et al. (2014). During a brief period of anesthesia with isoflurane (2%), 30 mg of 2,4-dinitrobenzenesulfonic acid (DNBS; Sigma-Aldrich, Milan, Italy) dissolved in 0.25 mL of 50% ethanol was intrarectally injected using a polyethylene PE-60 catheter inserted 8 cm proximal to the anus. Further, 0.25 mL of saline solution was injected in control rats.

### 2.3 Experimental design

Subgroups of animals with colitis were treated with a compound containing GABA-*Melissa officinalis* (Mo, dry extract leaves titrated at 2% in rosmarinic acid) preparation (GABA-Mo 5:1, Depofarma SpA, Italy, patent n. 102021000031112) or vehicle (1% carboxymethylcellulose), via oral gavage 80 mg/kg twice daily at the same time (8:00 a.m. and 18:00 p.m.). Two different experimental protocols of drug administration were adopted. In the preventive protocol, treatments started 3 days before DNBS and continued for further 14 days after colitis induction. In the curative protocol, treatments started 7 days after DNBS and continued for 14 days. Each protocol included four groups of treatment, arranged as follows:

• Group 1: Control + vehicle (n = 8)
• Group 2: DNBS + vehicle (n = 8)
• Group 3: Control + GABA-Mo (n = 8)
• Group 4: DNBS + GABA-Mo (n = 8)

**FIGURE 1:**
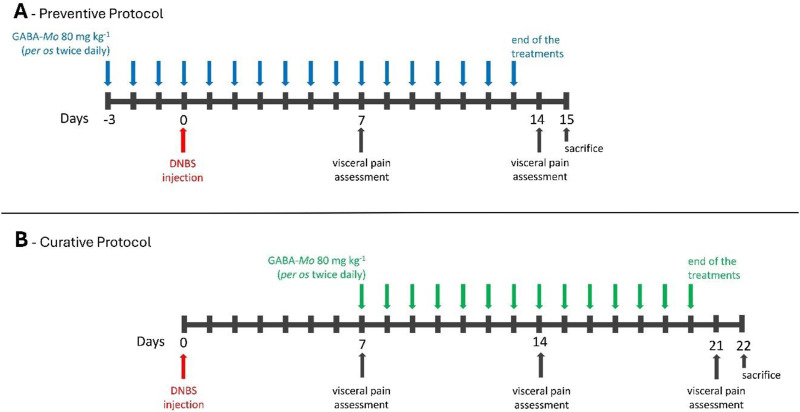
Schematic representation of the preventive **(A)** and curative **(B)** protocol used in the study.

As described in the experimental schemes in Figure 1, visceral pain assessments were performed on day 7 and 14 (both preventive and curative protocol) and on day 21 (curative protocol) after DNBS injection, 24 h after the last treatment with GABA-Mo or vehicle. Each protocol (preventive or curative) was performed in two experimental sets. Visceral pain assessments by the Abdominal Withdrawal Reflex (AWR) and Visceromotor Response (VMR) to colorectal distension (CRD) were performed on the same animals. Once visceral pain assessments were completed, animals were sacrificed to collect feces, blood samples, colonic tissues, and lumbar segments of spinal cord, which were processed to respectively assess the following parameters:

1) Microglia and astrocyte activation in the spinal cord
2) Macroscopic and histological damage to colon
3) Myeloperoxidase (MPO) and malondialdehyde (MDA) activity in colonic tissue
4) Tumor necrosis factor (TNF), interleukin (IL)-1β and IL-10 levels in plasma and colonic tissue
5) Occludin and claudin-1 expression in colonic tissue
6) Enteric glial cell activation in colonic tissue
7) Fecal calprotectin content
8) Plasma levels of Liposaccharide Binding Protein (LBP)

### 2.4 Assessment of visceral sensitivity by abdominal withdrawal response (AWR)

AWR to CRD was assessed by using a semi-quantitative score as described previously in conscious animals (Lucarini et al., 2020). Briefly, rats were anesthetized with isoflurane, and a lubricated latex balloon (length: 4.5 cm), attached to polyethylene tubing, assembled to an embolectomy catheter and connected to a syringe filled with water were inserted through the anus into the rectum and descending colon of adult rats. The tubing was taped to the tail to hold the balloon in place. Then rats were allowed to recover from the anaesthesia for 15 min. AWR measurement consisted of visual observation of animal responses to graded CRD (0.5, 1, 2, 3 mL) by a blinded observer who assigned the following scores: No behavioural response to colorectal distention (0); Immobile during colorectal distension and occasional head clinching at stimulus onset (1); Mild contraction of the abdominal muscles but absence of abdomen lifting from the platform (2); Observed strong contraction of the abdominal muscles and lifting of the abdomen off the platform (3); Arching of the body and lifting of the pelvic structures and scrotum (4).

### 2.5 Assessment of visceral sensitivity by viscero-motor reflex (VMR)

VMR to CRD was used as objective measure of visceral sensitivity. In animals under deep anaesthesia, two EMG electrodes were fixed into the external oblique abdominal muscle and exteriorized dorsally (Christianson and Gebhart, 2007). VMR assessment was carried out under light anesthesia (isoflurane 2%). A lubricated latex balloon (length: 4.5 cm), assembled to an embolectomy catheter and connected to a syringe filled with water was used to perform colon-rectal distension. A syringe was used to fill the balloon placed into the colon with various volumes of water (0.5, 1, 2, 3 mL). The electrodes were connected to a data acquisition system and the corresponding EMG signal consequent to colon-rectal stimulation was recorded, amplified, filtered, digitized, analysed and quantified using LabChart 8. To quantify the magnitude of the viscero-motor response at each distension volume, from the area under the curve (AUC) during the balloon distension (30 s) was subtracted the AUC immediately before the distension (30 s), and responses were expressed as percentage increase from the baseline. The time elapsed between two consecutive distensions was 5 min.

### 2.6 Immunofluorescence of the spinal cord

Spinal cords were fixed (paraformaldehyde 4%), exposed in succession to sucrose 20% and sucrose 30% (to prevent ice crystal formation in the tissues) and then snap frozen by using liquid nitrogen. The lumbosacral segment of the rat spinal cord was cut into 7 µm slices, rinsed three times (5 min each) in 1X phosphate-buffered saline (PBS), followed by a 1 h incubation in blocking solution (containing 0.1% Triton X-100, and 5% bovine serum albumin in 1X PBS) at room temperature. The slices were incubated overnight at 4°C with a rabbit anti-Ionized calcium binding adaptor molecule 1 (Iba-1, 1:500; Wako Chemicals, Richmond, VA, United States), diluted 1:250 in T-PBS/5% BSA (Sigma-Aldrich, Milan, Italy) or with a rabbit anti-glial fibrillary acidic protein (GFAP, DAKO-Z0334, Agilent Technologies Italia, Milan, Italy), diluted 1:500 in T-PBS/5% BSA (Sigma-Aldrich, Milan, Italy), respectively for microglia cells or for astrocytes staining. The following day, slides were washed thrice with PBS, and then incubated in blocking solution for 1 h with goat anti-rabbit IgG secondary antibodies (Invitrogen-Thermo Fisher Scientific, Milan, Italy) labelled with Alexa Fluor 488 for microglia and 647 for astrocytes. To stain the nuclei, sections were incubated with DAPI in PBS for 5 min at room temperature in the dark. After three washes in PBS and a final wash in distilled water, slices were mounted using Fluoromount-G™ Mounting Medium (Thermo Fisher Scientific, Milan, Italy) as mounting medium. Digitalized images were collected at ×200 total magnification using a motorized Leica microscope DM6 B equipped with a DFC9000 GT camera, supported by a THUNDER Workstation 3D DCV and by the software LAS X (Leica Biosystems, Milan, Italy). Quantitative analysis of GFAP- and Iba1-positive cells was performed by collecting at least three independent fields in the dorsal horns of each spinal cord and by using FIJI software (NIH, Bethesda, MD, United States). For each biomarker, primary antibodies were omitted for negative controls. Quantification of immunoreactivity was also performed using FIJI software by automatic thresholding images with the aid of an algorithm, which delivered the most consistent pattern recognition across all acquired images. The value relative to the background was subtracted from the value obtained from the analysed area, and the results were expressed as a percentage of the control group.

### 2.7 Macroscopic and microscopic damage

The evaluation of colonic macroscopic damage was performed in accordance with the criteria and score reported previously (Khan et al., 2017): presence of adhesions between colon and other intra-abdominal organs (0–2); consistency of colonic fecal material (0–2); thickening of colonic wall (mm); presence and extension of hyperemia and macroscopic mucosal damage (0–5).

Microscopic evaluation was carried out on hematoxylin/eosin-stained sections of full-thickness samples obtained from the distal colon, fixed in 4% paraformaldehyde, paraffin embedded, and sectioned in 5 µm slices. The microscopic damage was scored in accordance with the criteria reported previously (Morris et al., 1989): mucosal architecture loss (0–3); goblet cell depletion (0, absent; 1, present); crypt abscess (0, absent; 1, present); cellular infiltration (0–3); tunica muscularis thickening (0–3).

### 2.8 Evaluation of tissue malondialdehyde levels

MDA concentration in intestinal tissues was determined to obtain quantitative estimates of membrane lipid peroxidation (Fornai et al., 2014). For this purpose, intestinal tissue was excised, weighed, minced by forceps, homogenized in 2 mL of cold buffer (Tris-HCl 20 mM, pH 7.4) using a Polytron homogenizer (QIAGEN, Milan, Italy), and centrifuged at 1500 g for 10 min at 4°C. Aliquots of supernatants were then used for subsequent assay procedures. Tissue MDA concentrations were estimated using a colorimetric assay kit (Cayman Chemical, Ann Arbor, MI, U.S.A.). The results were expressed as nmoles of MDA per milligram of intestinal tissue.

### 2.9 Evaluation of tissue myeloperoxidase levels

MPO was assumed as a quantitative index to estimate the degree of mucosal infiltration by polymorphonuclear cells (Fornai et al., 2014). Specimens of colon (30 mg) were homogenized on ice with a Polytron homogenizer (QIAGEN, Milan, Italy) in 0.6 mL of ice-cold lysis buffer (200 mM NaCl, 5 mM EDTA, 10 mM Tris, 10% glycerine, 1 mM phenylmethylsulfonylfluoride (PMSF), 1 μg/mL leupeptin and 28 μg/mL aprotinin, pH 7.4). The homogenates were centrifuged 2 times at 4°C for 15 min at 1500 g. The supernatants were diluted 1:5 and used for determination of MPO concentration by means of enzyme-linked immunosorbent assay (ELISA) (Hycult Biotech, Uden, Netherlands). All samples were assayed within 2 days after collection. The results were expressed as nanograms of MPO per milligram of intestinal tissue.

### 2.10 Evaluation of TNF, IL-1β and IL-10 levels in plasma and colonic tissue

TNF, IL-1β and IL-10 levels in plasma and colon were measured by ELISA kits, as previously described (Pellegrini et al., 2016). For colonic tissue the following ELISA kits were used: TNF (Thermo Fisher, ERA57RB), IL-1β (Abcam, ab100768) and IL-10 (Thermo Fisher ERA24RB). For this purpose, tissue samples (20 mg), stored previously at −80 °C, were weighed, thawed, and homogenized in 0.4 mL of PBS (pH 7.2) and centrifuged at 10,000 g for 5 min at 4°C. The following ELISA kits were used for the measurement of the cytokines in plasma: TNF (Invitrogen, 88734022), IL-1β (Abcam, ab100767) and IL-10 (Abcam, ab-100764).

For the procedure, blood samples were centrifuged for 5 min at 4000 g at 2°C–8°C. After centrigufation supernatants were collected. Aliquots (100 μL) of supernatants were used for assay. Tissue and plasma cytokine levels were expressed as ρg/mg of tissue and ρg/mL of plasma, respectively.

### 2.11 Assay of fecal calprotectin

Fecal pellets were frozen dried for 24 h and reconstituted in 1 mL PBS, along with 50 μL 1% (wt/vol) ascorbic acid (Sigma, St Louis, MO, United States). Samples were then homogenized for 10 min (4°C). Homogenates were diluted with 2 mL lysis buffer (0.1% sodium dodecylsulfate, 0.5% sodium deoxycholate, 0.02% sodium azide, 5 mM disodium ethylenediaminetetraacetic acid, and 1× Halt protease/phosphatase inhibitor cocktail [Thermo Fisher Scientific Inc, Waltham, MA, United States] in PBS). Homogenates were further homogenized for 30 s and centrifuged (5800 g, 10 min, 4°C), and supernatants were snap frozen in liquid nitrogen and stored at −80°C. Fecal calprotectin levels were determined using a rat calprotectin ELISAkit (Cusabio EQ013485RA). Homogenates were diluted 2-fold with PBS and analysed following the manufacturer’s instructions. Results were expressed as nanograms of calprotectin per milligram of feces.

### 2.12 Western blot assay of colonic occludin, claudin-1 and S-100β

Colonic tissue samples were collected and flushed of fecal content with ice-cold PBS, as described previously (Antonioli et al., 2021). Tissues were minced and homogenized using a Potter-Elvehjem Grinder homogenizer on ice in 20% (w/v) TNE lysis buffer (50 mM Tris-HCl pH 7.4, 100 mM NaCl, 0.1 mM EDTA, 1% NP-40, 1% SDS, 0.1% DOC) with proteases and phosphatases inhibitors. Samples were then sonicated and boiled for 5 min at 95°C. Proteins were quantified with the Bradford assay. Proteins (30 µg) were separated onto a pre-cast 4%–20% polyacrylamide gel (Mini-PROTEAN^®^ TGX gel, Biorad) and then transferred to PVDF membranes (Trans-Blot^®^ TurboTM PVDF Transfer packs, Biorad). Membranes were blocked with 3% BSA diluted in Tris-buffered saline (TBS, 20 mM Tris-HCl, pH 7.5, 150 mM NaCl) with 0.1% Tween 20. Primary antibodies against β-actin (Sigma, A3854), claudin-1 (Abcam, ab180158), occludin (Acam, ab167161) and S100-β (Abcam, ab52642) were used. Secondary antibodies were obtained from Abcam (anti-mouse ab97040 and anti-rabbit ab6721). Protein bands were detected with ECL reagents (Clarity-Western ECL Blotting Substrate, Biorad). Densitometry was performed by IBright Analysis software.

### 2.13 Evaluation of plasma LBP

Plasma LBP levels were quantified using a commercial ELISA kit (Cusabio, CSB-E11184r). For the procedure, blood samples were centrifuged for 5 min at 4000 g at 2°C–8°C. After centrifugation supernatants were collected. For the assay, aliquots (100 µL) of plasma were used. LBP concentrations were expressed as picograms per millilitre (ρg/mL).

### 2.14 Statistical analysis

The statistical analysis of data complies with the requirements of good laboratory practices (GLP). Results were presented as mean ± standard error of the mean (S.E.M.). All the group sizes were designed to be homogeneous. The analysis of variance of visceral pain assessments and histological data were performed by one-way ANOVA with Bonferroni’s significant difference procedure used for post-hoc comparisons. P values of less than 0.05 were considered significant. Data were analyzed using the “Origin 9” software (OriginLab, Northampton, United States of America). The statistical significance of molecular data was evaluated by one-way analysis of variance (ANOVA) followed by post hoc analysis by with Dunnet or Tukey test where appropriate, and P values lower than 0.05 were considered significant. All statistical procedures were performed using GraphPad Prism 9.0 software (GraphPad Prism, San Diego, CA, United States).

## 3 Results

### 3.1 Effect of GABA-Mo on the development and persistence of visceral hypersensitivity resulting from colitis in rats

**FIGURE 2:**
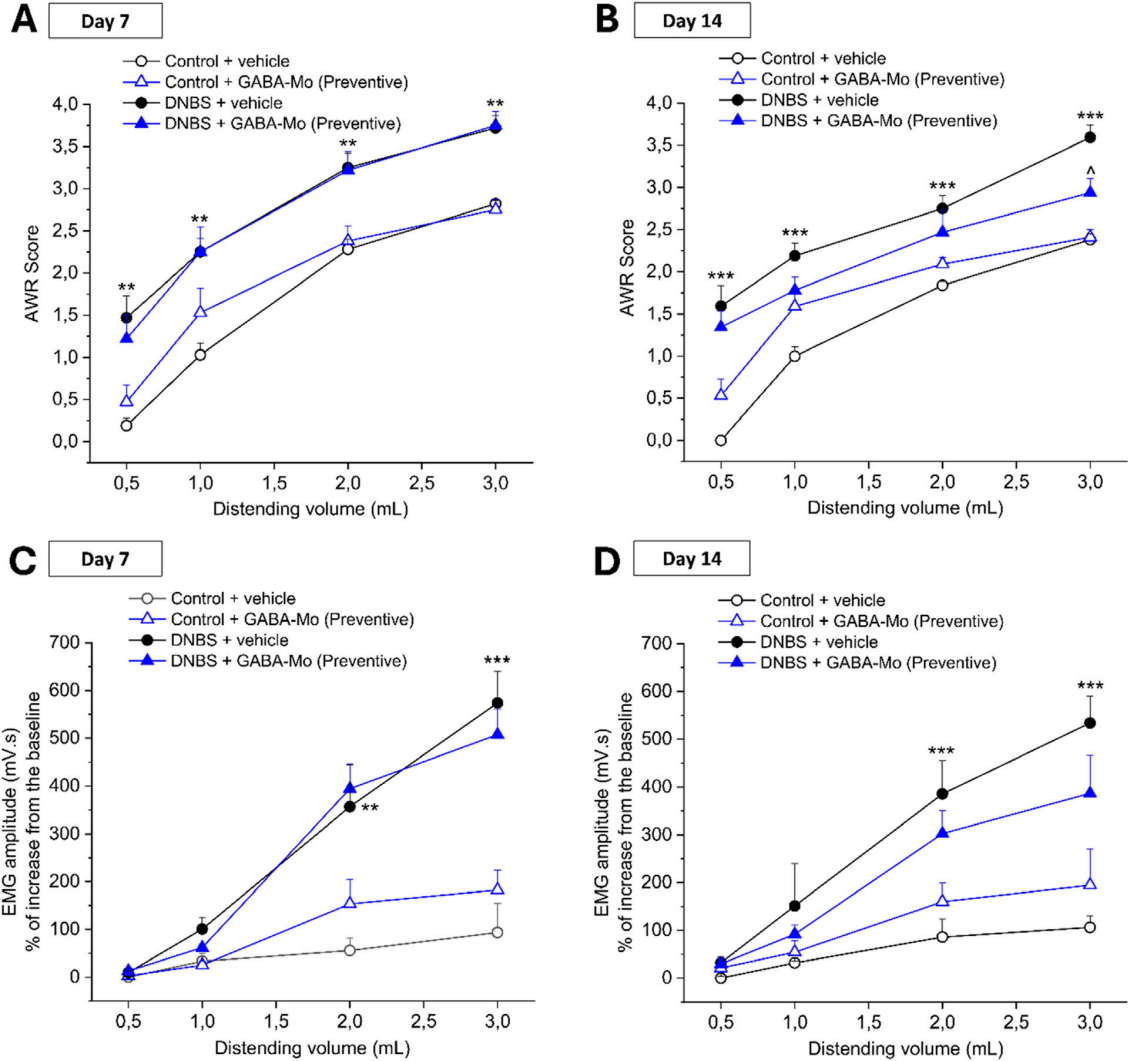
Effect of the preventive protocol of treatment with GABA-Mo on visceral pain induced by DNBS in rats. Animal nocifensive response associated with visceral pain was assessed by measuring the extent of the abdominal withdrawal response (AWR) to colorectal distension (CRD; 0.5–3 mL; A-B). Animal visceral sensitivity was assessed by measuring the extent of the abdominal contraction (EMG amplitude) related to visceromotor response (VMR) to colorectal distension (CRD; 0.5–3 mL; C-D). The test was performed 7 days **(A,C)** and 14 days **(B,D)** after colitis induction. The test was performed 7 days **(A)** and 14 days **(B)** after colitis induction. *P < 0.05, **P < 0.01 and ***P < 0.001 vs Control + vehicle treated animals. ^∧^ *P* < 0.05 vs DNBS+ vehicle treated animals.

**FIGURE 3:**
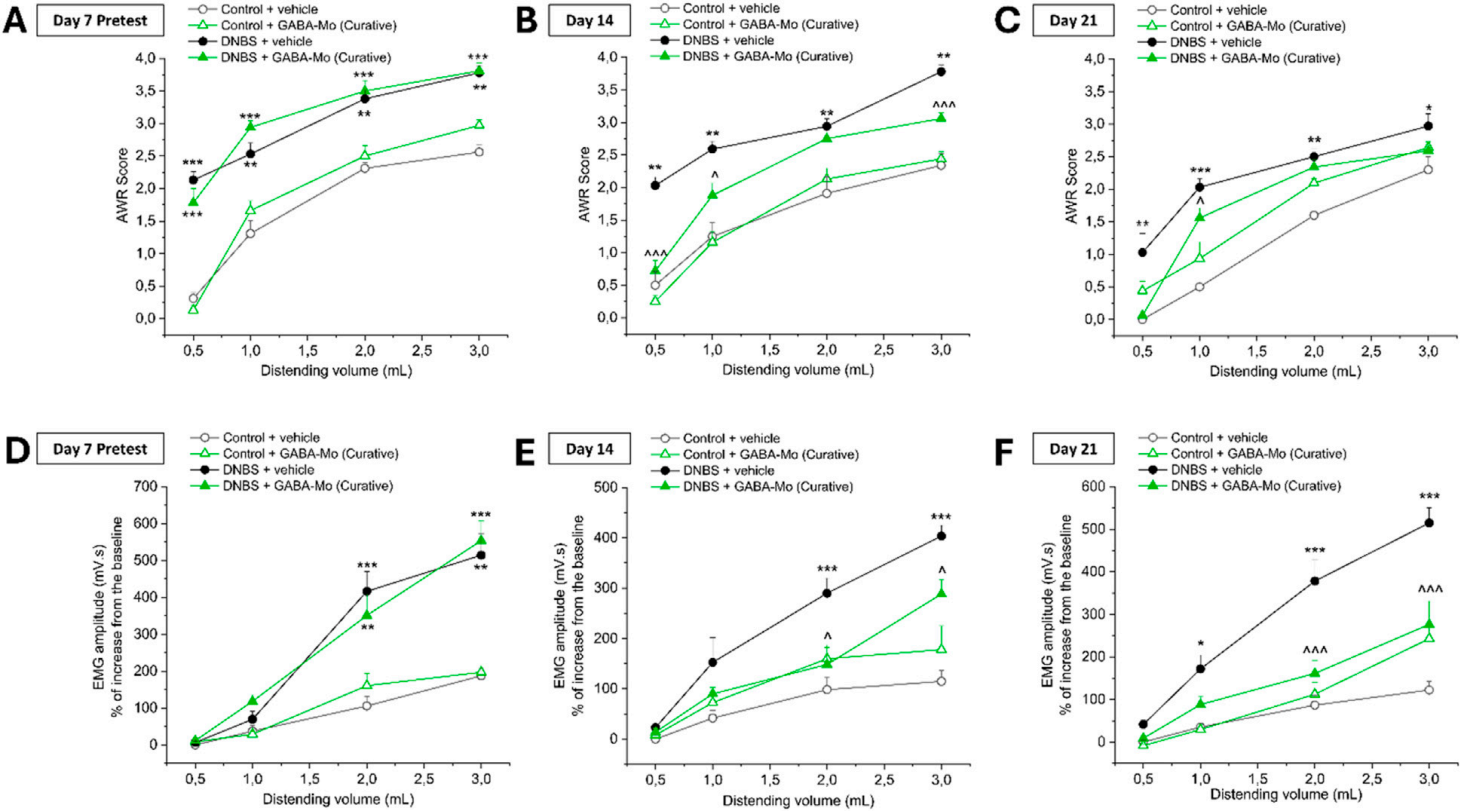
Effect of the curative protocol of treatment with GABA-Mo on visceral pain induced by DNBS in rats. Animal nocifensive response associated with visceral pain was assessed by measuring the extent of the abdominal withdrawal response (AWR) to colorectal distension (CRD; 0.5–3 mL; A-C). Animal visceral sensitivity was assessed by measuring the extent of the abdominal contraction (EMG amplitude) related to visceromotor response (VMR) to colorectal distension (CRD; 0.5–3 mL; D-E). The tests were performed 7 days [(**A,D)**, pretest], 14 days **(B,E)** and 21 days **(C,F)** after colitis induction. **P < 0.01 and ***P < 0.001 vs. Control + vehicle treated animals. ^∧^ *P* < 0.05, ^∧^^∧^ *P* < 0.01 and ^∧^^∧^^∧^ *P* < 0.001 vs. DNBS + vehicle treated animals.

Figures 2, 3 show the effect of the administration of GABA-Mo on visceral hypersensitivity induced by DNBS intrarectal injection in rats. Visceral pain was assessed in rats by measuring the Abdominal Withdrawal Reflex (AWR, Figures 2A, B; Figures 3A–C) and the Viscero-Motor Response (VMR, Figures 2C, D; Figures 3D–F) to colorectal distension (0.5–3 mL). In the preventive protocol, the nocifensive behaviour of animals was measured by assigning a score (0–4) to their AWR in response to colorectal distension (0.5–3 mL) at day 7 (acute inflammatory phase) and 14 (post-inflammatory phase) for the preventive protocol (Figures 2A, B, respectively) and at baseline (day 7) and at day 14 and 21 (post-inflammatory phase) for the curative protocol (Figures 3A–C, respectively). The AWR score of control animals was constant over time and directly proportional to the applied stimulus. In both preventive and curative protocols, DNBS-treated animals displayed a significantly higher abdominal response than controls at each distending volume tested. Indeed, the response of DNBS + vehicle group was significantly increased either at day 7, 14 or 21 in both the experimental paradigms (Figures 2, 3). The preventive treatment with GABA-Mo reduced AWR score in DNBS animals only after 14 days, though the statistical significance was reached only with the highest distending volume (3 mL) (Figure 2B). On day 7, before starting the curative GABA-Mo treatment, all DNBS treated animals displayed a higher nocifensive response to CRD than controls (Figure 3A). The curative administration of GABA-Mo significantly ameliorated AWR scores both on day 14 and 21, for the distending volumes of 0.5, 1 and 3 mL and 0.5 and 1 mL, respectively (Figures 3B, C). Besides, animal visceral sensitivity was assessed by measuring the VMR to colorectal distension (0.5–3 mL) at day 7 and 14 for the preventive protocol (Figures 2C, D) and at baseline (day 7) and at day 14 and 21 for the curative protocol and (Figures 3D–F). The induction of colitis was associated with a remarkable and persistent increase in visceral sensitivity, statistically significant either on day 7, 14 or 21. The amplitude of the abdominal contraction, assessed with VMR test, was directly proportional to the applied stimulus. DNBS-treated animals displayed a higher VMR than controls at 1–3 mL distending volumes, both in the preventive and curative protocols (Figures 2, 3, respectively). The preventive administration of GABA-Mo did not exert significant effects on DNBS-induced VMR increase, though a slight reduction of EMG amplitude can be detected on day 14 with the treatment (Figure 2D). On the other hand, GABA-Mo administered according to the curative protocol significantly reduced VMR both after 14 and 21 days, an effect which resulted statically significant for the distending volumes of 2 and 3 mL (Figures 3E, F). Although visceral sensitivity of Control + GABA-Mo group resulted slightly increase with respect of Control + vehicle group in both AWR (Figures 2A, B; Figures 3A–C) and VMR (Figure 2C, D; Figure 3D–F) assessments, the overall effect of the treatment on normal sensitivity did not result statistically significant.

### 3.2 Effect of GABA-Mo on microglia and astrocytes activation in the spinal cord resulting from colitis in rats

**FIGURE 4:**
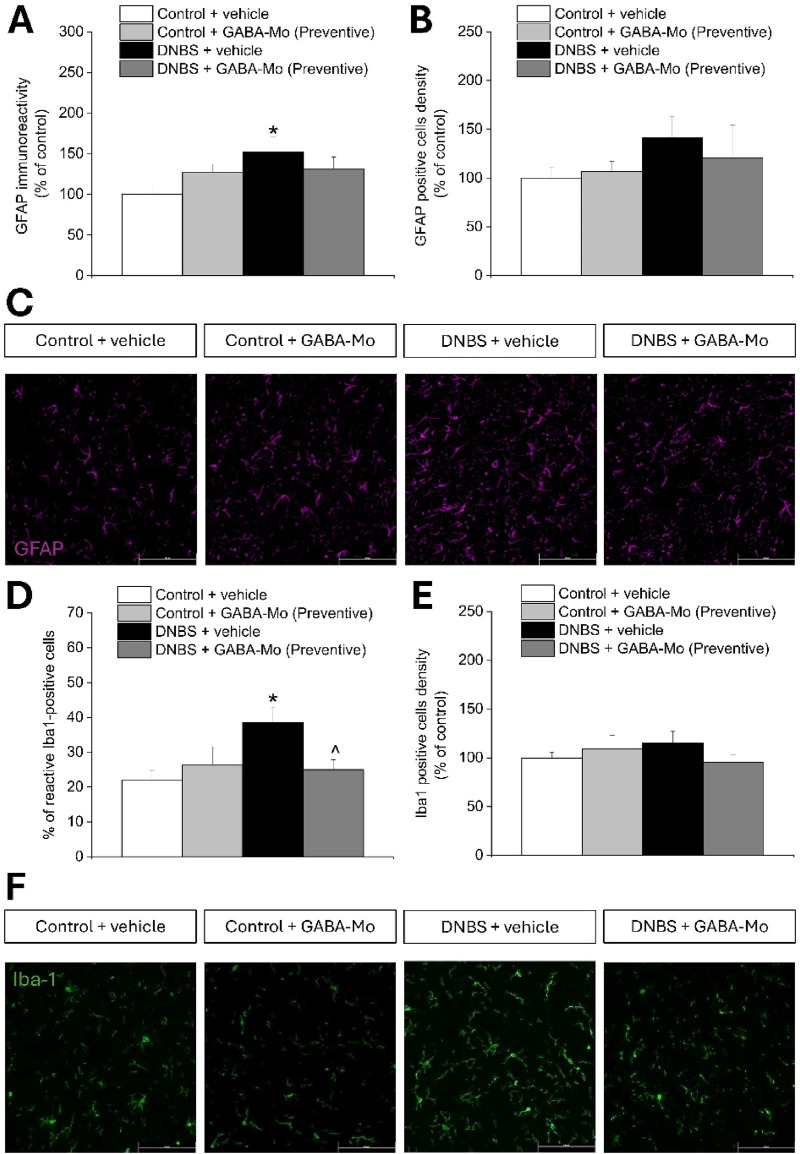
Effect of DNBS treatment and preventive protocol with GABA-Mo on astrocytes and microglia activation in the spinal cord. The graphs show the quantification of GFAP immunoreactivity **(A)** and the density of GFAP-positive cells **(B)**, with representative images showing the expression of GFAP (purple); **(C)**, 14 days after DNBS injection. The figure reports the quantification of the percentage of reactive Iba-1-positive cells **(D)** and the density of Iba-1-positive cells **(E)**, with representative images showing the expression of Iba-1 (green); **(F)**, 14 days after DNBS injection. The quantitative analysis of immunofluorescence was performed by collecting independent fields from the dorsal horns of the spinal cord. *P < 0.05 vs. Control + vehicle group. ^∧^ *P* < 0.05 vs. DNBS + vehicle group. Original magnification: ×20.

**FIGURE 5:**
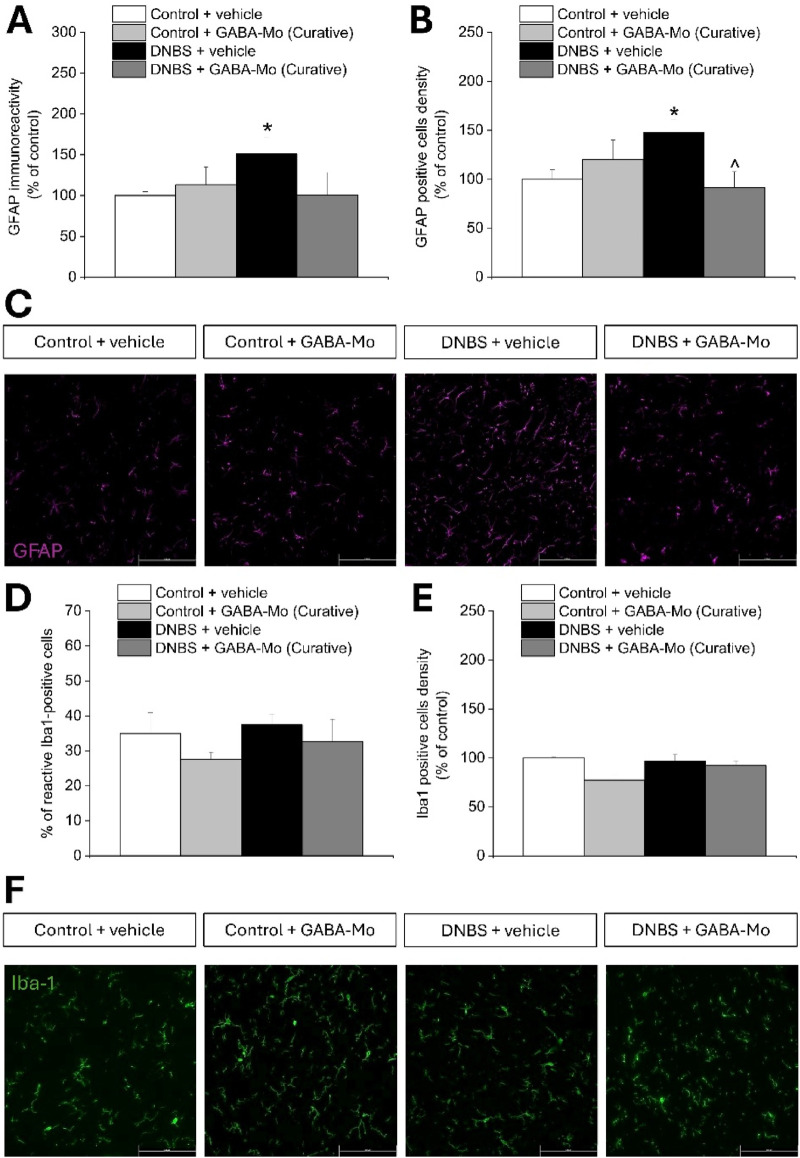
Effect of DNBS treatment and curative protocol with GABA-Mo on astrocytes and microglia activation in the spinal cord. The graphs show the quantification of GFAP immunoreactivity **(A)** and the density of GFAP-positive cells **(B)**, with representative images showing the expression of GFAP (purple); **(C)**, 21 days after DNBS injection. The figure reports the quantification of the percentage of reactive Iba-1-positive cells **(D)** and the density of Iba-1-positive cells **(E)**, with representative images showing the expression of Iba-1 (green); **(F)**, 14 days after DNBS injection. The quantitative analysis of immunofluorescence was performed by collecting independent fields from the dorsal horns of the spinal cord. *P < 0.05 and **P< 0.01 vs. Control + vehicle group. ^∧^^∧^ *P*< 0.01 vs. DNBS + vehicle group. Original magnification: ×20.

Figures 4, 5 show the morphology and the density of astrocytes (GFAP-positive cells) and microglial cells (Iba1-positive cells) on sections of lumbo-sacral spinal cord of GABA-Mo treated animals 14 or 21 days after DNBS injection (according to the preventive and curative protocol, respectively), evaluated by immunofluorescence analysis after completing the behavioural tests. On day 14 (preventive protocol), DNBS-treated rats showed a higher GFAP-related immunoreactivity compared to control group (Figure 4A), though the density of GFAP-positive cells did not significantly augment (Figure 4B), indicating an increased reactivity of astrocytes, as it emerges from the representative images (Figure 4C). Supplementation with GABA-Mo by preventive protocol did not significantly influence GFAP immunoreactivity or GFAP-positive cells density in DNBS-treated animals (Figures 4A–C). At the same time, the percentage of reactive Iba-1 positive cells was significantly higher in DNBS-treated animals as compared to controls, though their density in the tissue did not change (Figures 4D, E). In this setting, GABA-Mo determined a normalization of the percentage of reactive Iba-1-posititve cells which was comparable to that of controls (Figure 4D), without affecting the cellular density (Figure 4E), as it emerges from the representative images (Figure 4F). The activated status of microglial cells was recognized by the loss of the processes that are peculiar of resting conditions (Fernández-Arjona et al., 2017). On day 21 (curative protocol), astrocytes from DNBS-treated rats showed both a higher immunoreactivity and density compared to control group (Figures 5A, B), as it is attested by the representative images (Figure 5C). Supplementation with GABA-Mo significantly reduced GFAP positive cells density (Figure 5B) and slightly lowered GFAP immunoreactivity, even though this effect did not reach the statical significance (Figure 5A). In the same experimental setting, microglia (Iba1-positive cells) did not change in either density or reactivity in any group (Figures 5D–F). GABA-Mo administration did not influence astrocytes (Figures 4A–C, 5A–C) and microglia (Figures 4D–F, 5D–F) immunoreactivity and density in the spinal cord of controls, as it was observed in both experimental paradigms (preventive and curative protocol).

### 3.3 GABA-Mo effect on colon macroscopic and microscopic damage resulting from colitis in rats

**FIGURE 6:**
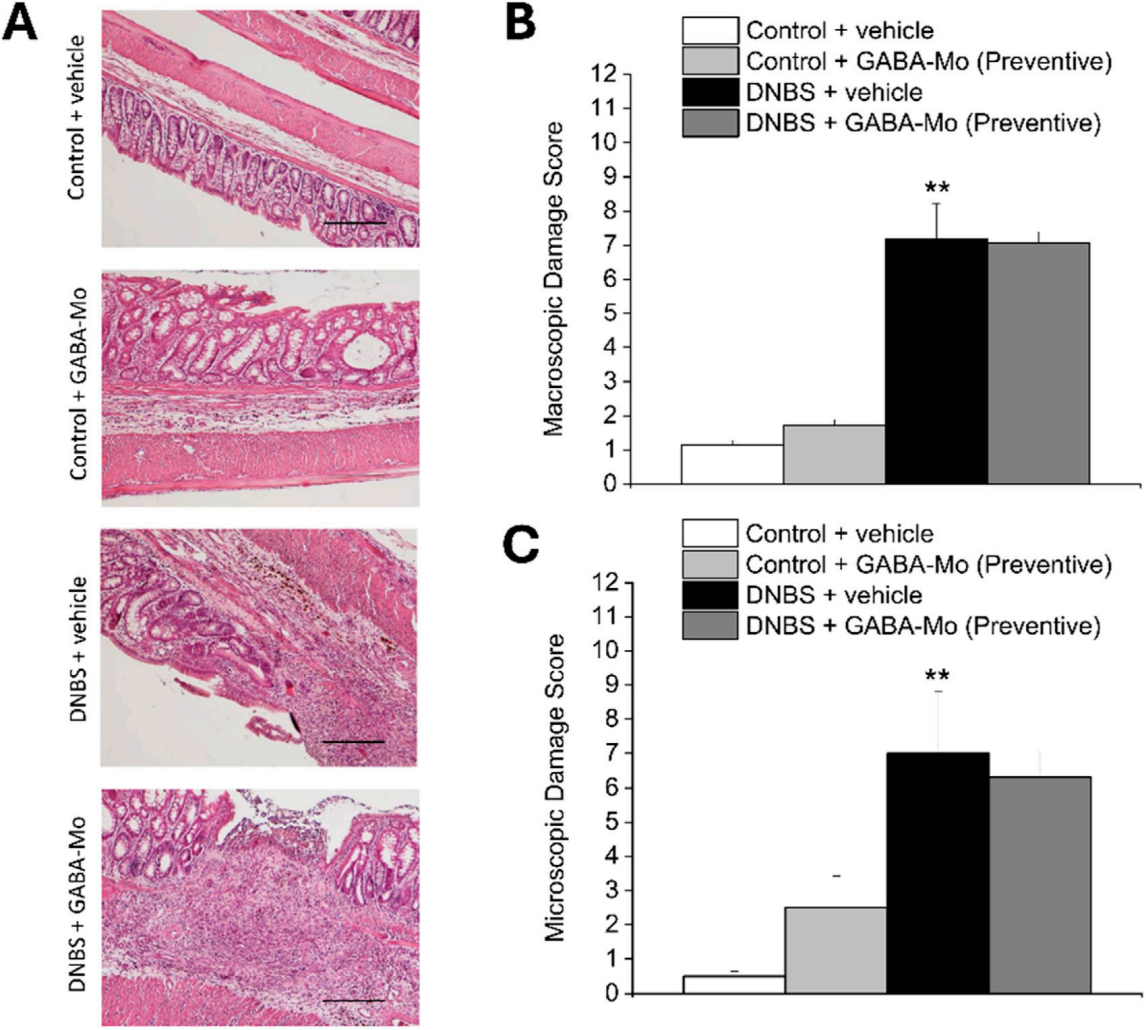
Effect of preventive protocol with GABA-Mo on colon damage induced by DNBS in rats. Representative pictures of haematoxylin–eosin-stained sections of full-thickness colon were reported **(A)**; Original magnification ×10). The column graphs report the colon macroscopic **(B)** and microscopic **(C)** damage score; **P < 0.01 vs. Control + vehicle.

**FIGURE 7:**
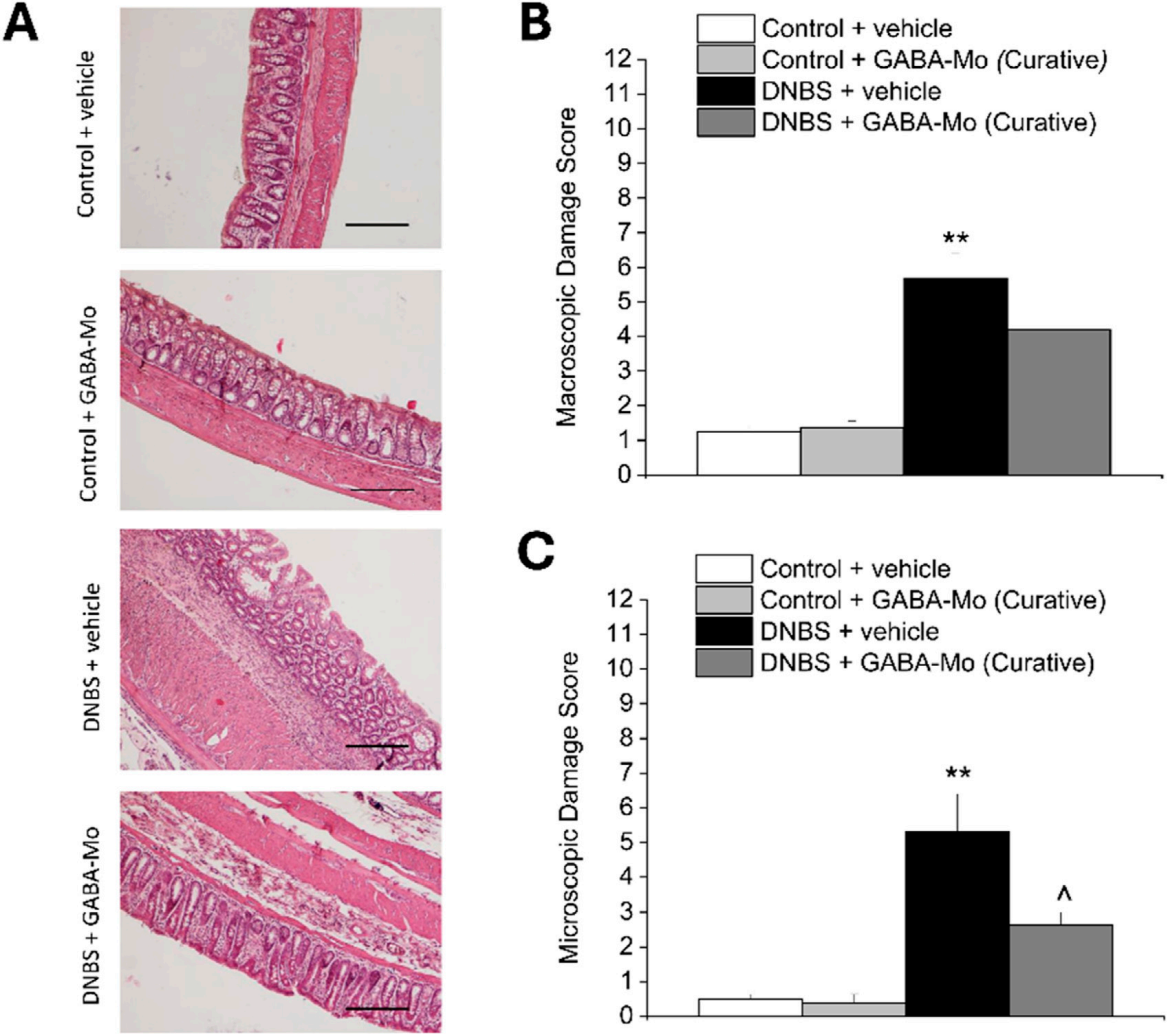
Effect of curative protocol with GABA-Mo on colon damage induced by DNBS in rats. Representative pictures of haematoxylin–eosin-stained sections of full-thickness colon were reported **(A)**; Original magnification ×10). The column graphs report the colon macroscopic **(B)** and microscopic **(C)** damage score; **P < 0.01 vs. Control + vehicle. ^∧^ *P* < 0.05 vs. DNBS + vehicle.

As depicted in the representative images of Haematoxylin/Eosin-stained sections of full-thickness colon (Figure 6A), the animals sacrificed on day 14 after DNBS injection (preventive protocol) showed a significant macroscopic and microscopic damage compared with control group (Figures 6B, C, respectively). The preventive treatment with GABA-Mo did not exert any relevant effect on DNBS-induced colon damage, regarding both scores (Figures 6B, C). Animals sacrificed 21 days after DNBS injection (curative protocol), displayed a still significant increase in colonic damage score, both macroscopically and microscopically with respect of control group (Figures 7A–C). Anyway, in this experimental setting, GABA-Mo treatment significantly ameliorated colon damage both macroscopically and microscopically (Figures 7B, C, respectively), though only the score related to the microscopic damage resulted significantly reduced with respect of DNBS + vehicle group (Figure 7C). Looking at H/E-stained sections (Figure 7A), after the curative treatment with GABA-Mo, the colonic wall of DNBS animals appeared significantly less thick in comparison to that of DNBS + vehicle animals, with reduced inflammatory infiltrate and oedema in the submucosae and an almost completely restored mucosa (Figure 7A). With both protocols of administration (Figures 6, 7, preventive and curative protocol, respectively), GABA-Mo did not alter colon histology of control animals.

### 3.4 Evaluation of tissue myeloperoxidase and malondialdehyde levels

**FIGURE 8:**
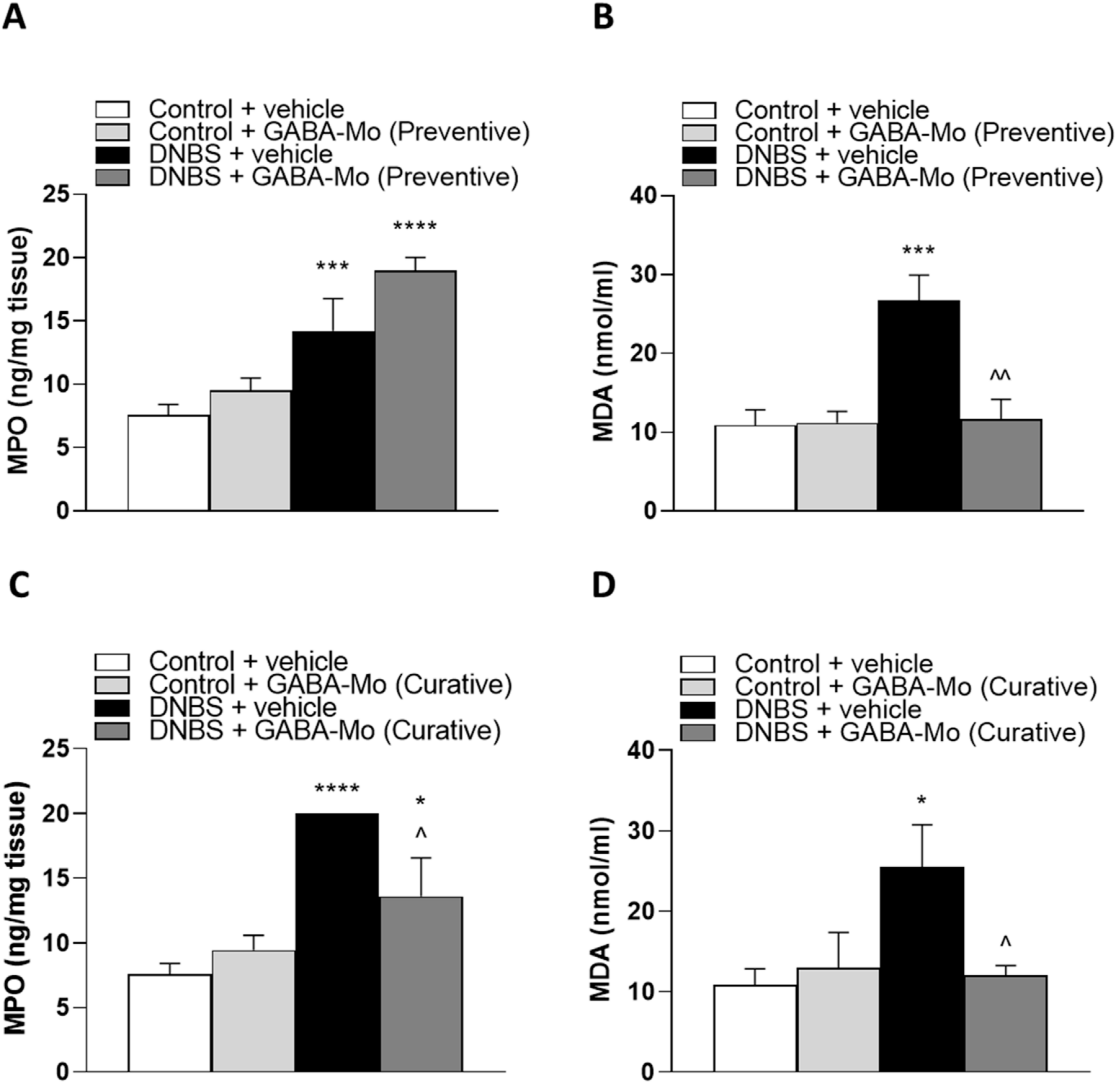
Colonic levels of MPO **(A)** and MDA **(B)** in the preventive protocol and MPO **(C)** and MDA **(D)** in the curative protocol in control or DNBS rats treated with vehicle or GABA-Mo. Each column represents the mean ± SEM from eight animals. *P < 0.05, ***P < 0.001, ****P < 0.0001 vs. Control + vehicle group. ^∧^ *P* < 0.05 and ^∧^^∧^ *P* < 0.01 vs. DNBS + vehicle group.

In the preventive protocol, tissue MPO, index of mucosal infiltration by polymorphonuclear cells, and MDA, marker of oxidative stress, were significantly higher in colonic tissues from DNBS animals (Figures 8A, B) as compared with control animals. The supplementation with GABA-Mo significantly lowered MDA levels but showed no effects on MPO levels (Figures 8A, B).

In the curative protocol, both MPO and MDA levels were significantly increased in DNBS animals in comparison with control animals (Figures 8C, D). In this setting, the administration of GABA-Mo significantly reduced both parameters (Figures 8C, D).

### 3.5 Evaluation of TNF, IL-1β and IL-10 levels in plasma and colonic tissue

**FIGURE 9:**
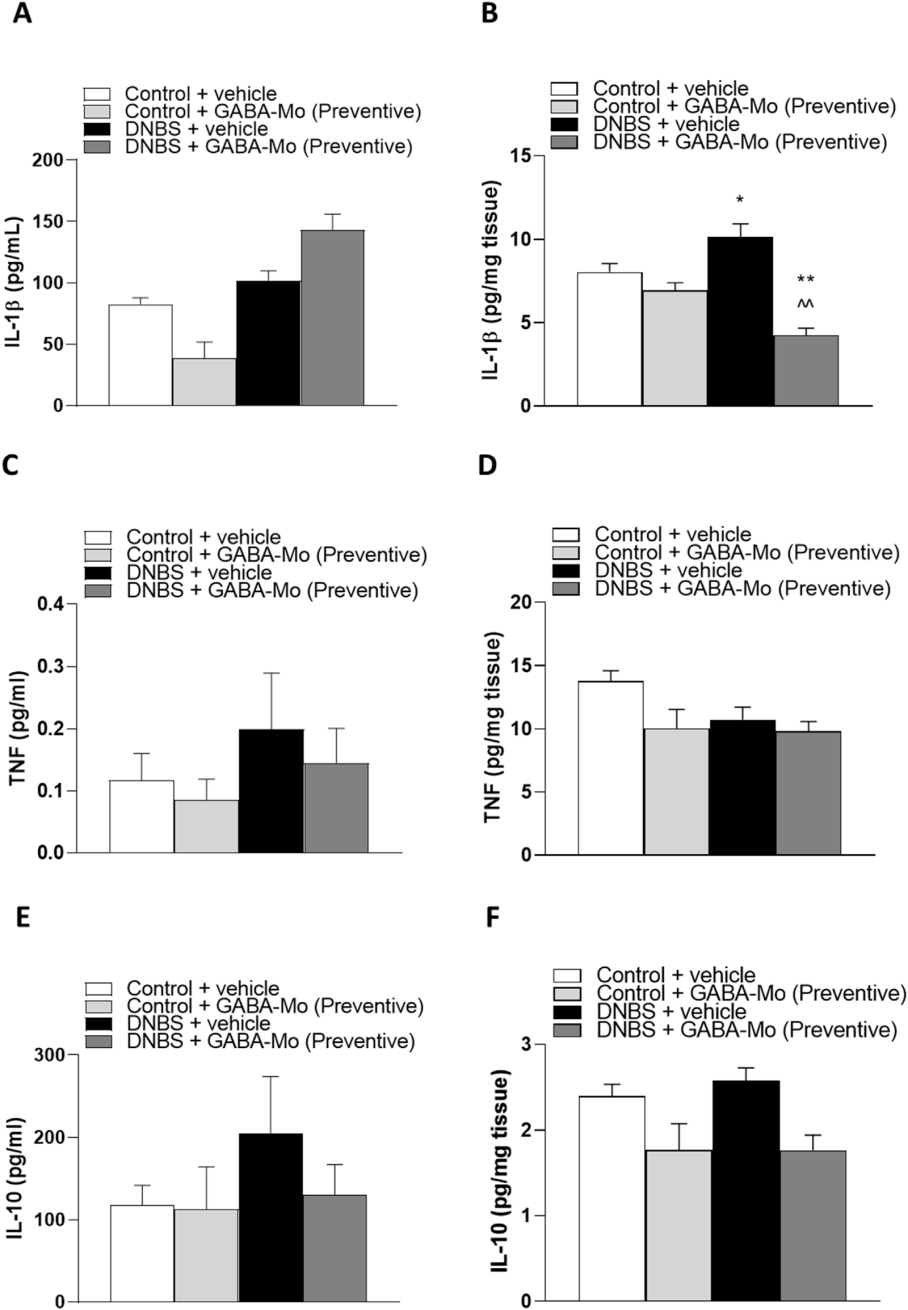
Preventive protocol. IL-1β levels in plasma **(A)** and colonic tissues **(B)**; TNF levels in plasma **(C)** and colonic tissues **(D)**, IL-10 levels in plasma **(E)** and colonic tissues **(F)** in control or DNBS rats treated with vehicle or GABA-Mo. Each column represents the mean ± SEM from eight animals. **P < 0.01 vs. Control + vehicle group. ^∧^^∧^ *P* < 0.01 vs. DNBS + vehicle group.

In the preventive protocol, IL-1β plasma levels showed no significant alterations in all treatment groups (Figure 9A). In contrast, IL-1β colonic levels were significantly increased in DNBS animals. In this context, treatment with GABA-Mo significantly reduced IL-1β colonic levels, as compared to both controls and DNBS rats (Figure 9B). Both plasma and colonic levels of TNF and IL-10 were not significantly changed by all treatments (Figures 9C–F).

**FIGURE 10:**
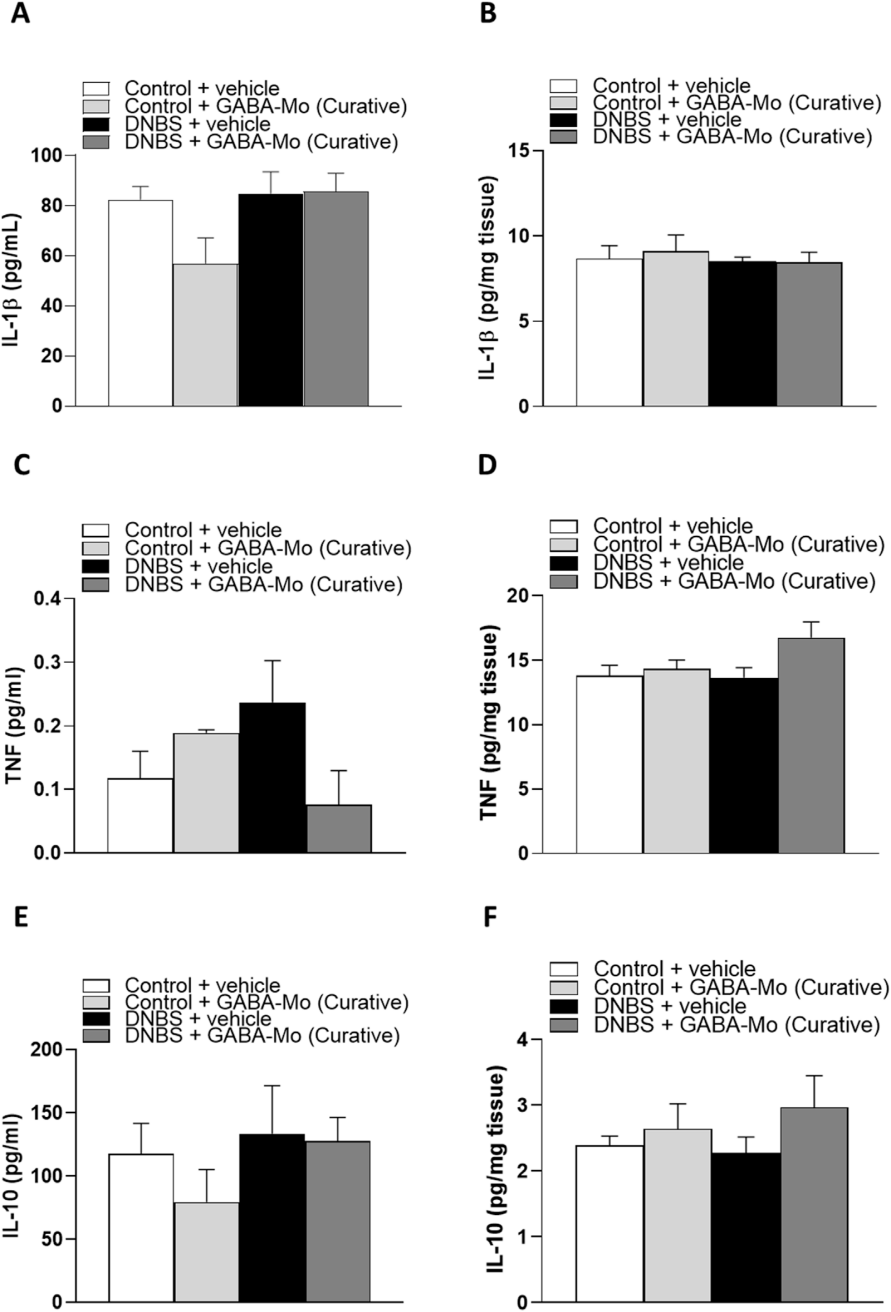
Curative protocol. IL-1β levels in plasma **(A)** and colonic tissues **(B)**; TNF levels in plasma **(C)** and colonic tissues **(D)**, IL-10 levels in plasma **(E)** and colonic tissues **(F)** in control or DNBS rats treated with vehicle or GABA-Mo. Each column represents the mean ± SEM from eight animals.

In the curative protocol, no significant alterations of IL-1β, TNF or IL-10 levels were detected in all experimental groups (Figures 10A–F).

### 3.6 Assay of fecal calprotectin

**FIGURE 11:**
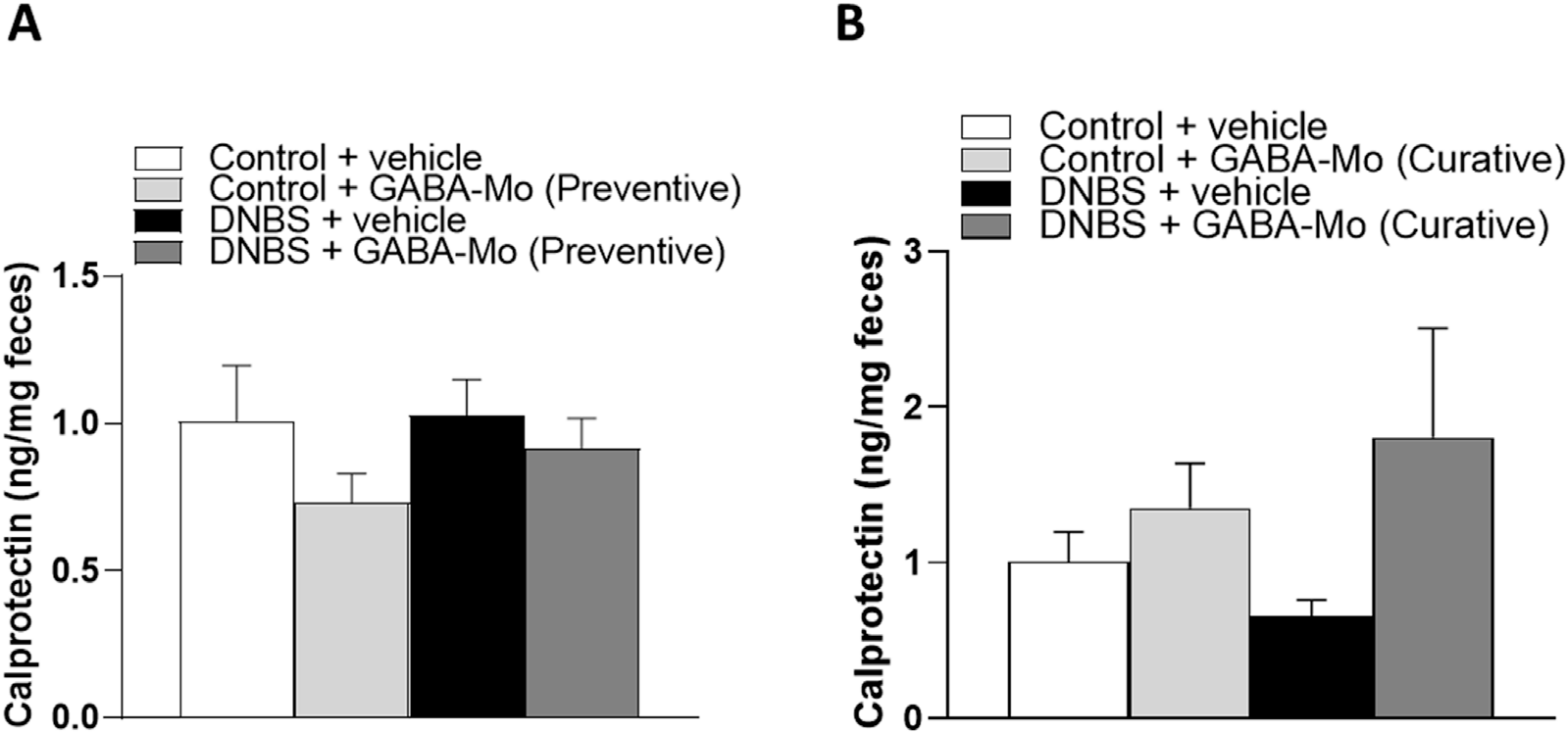
Calprotectin levels in the preventive protocol of treatment **(A)** and curative protocol of treatment **(B)** in feces from control or DNBS rats treated with vehicle or GABA-Mo. Each column represents the mean ± SEM from eight animals.

Administration of GABA-Mo did not significantly alter fecal calprotectin levels in both preventive and curative protocols (Figures 11A, B).

### 3.7 Western blot assay of colonic S-100β, occludin and claudin-1

**FIGURE 12:**
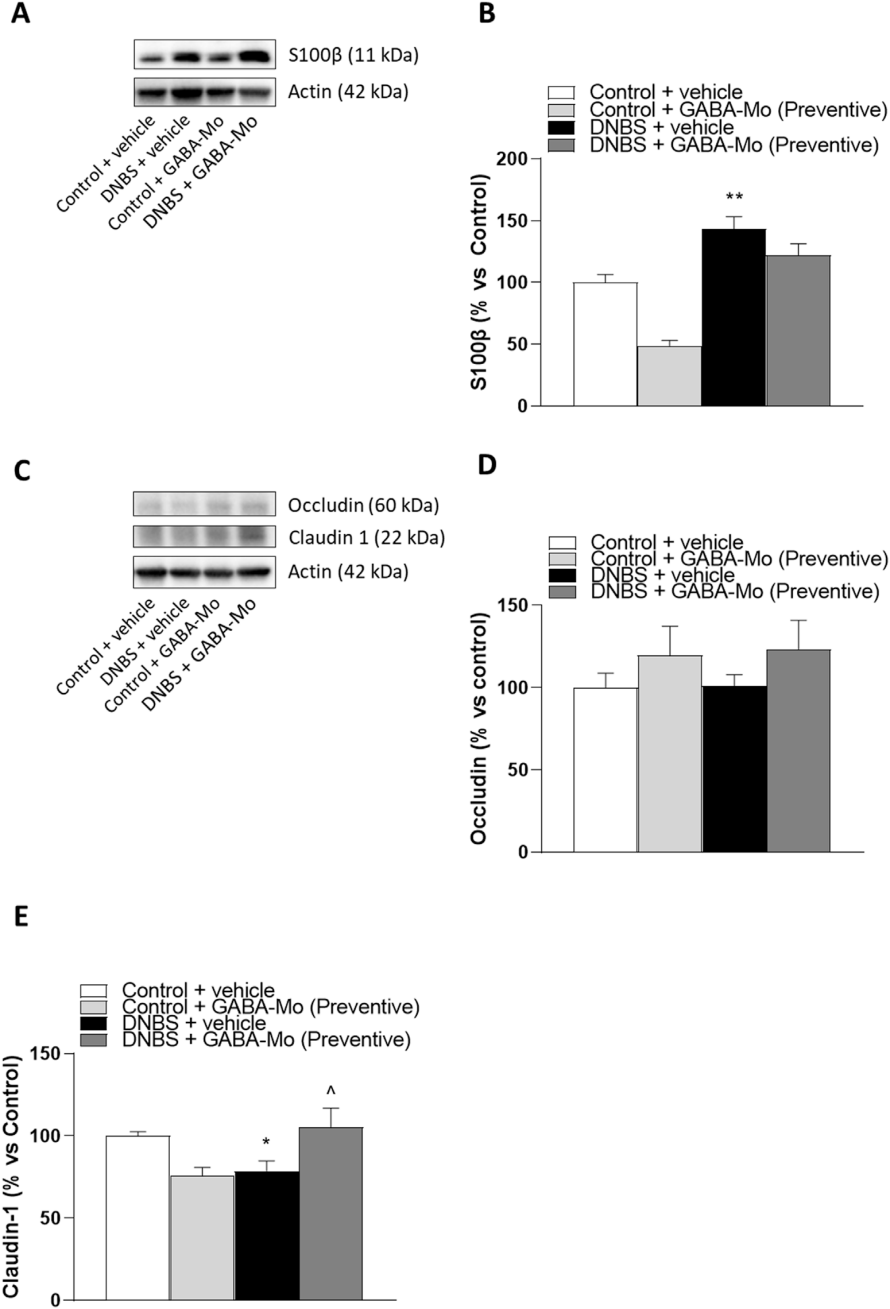
Preventive protocol. Representative blots **(A and C)** and densitometric analysis of the expression levels of S100β **(B)**, occludin **(D)** and claudin-1 **(E)** in control or DNBS rats treated with vehicle or GABA-Mo. Each column represents the mean ± SEM from eight animals. *P < 0.05, **P < 0.01 vs. Control + vehicle group. ^∧^ *P* < 0.05 vs. DNBS + vehicle group.

In the preventive protocol, DNBS-animals showed a significant increase in S100β expression as compared to controls (Figures 12A, B). In this setting GABA-Mo did not exert ameliorative effects. The expression of occludin in colonic tissues was not significantly changed in all treatment groups (Figures 12C, D), whereas claudin-1 expression was significantly lower in DNBS-treated rats, as compared with control animals (Figures 12C, E). The supplementation with GABA-Mo normalised claudin-1 expression (Figures 12C–E).

**FIGURE 13:**
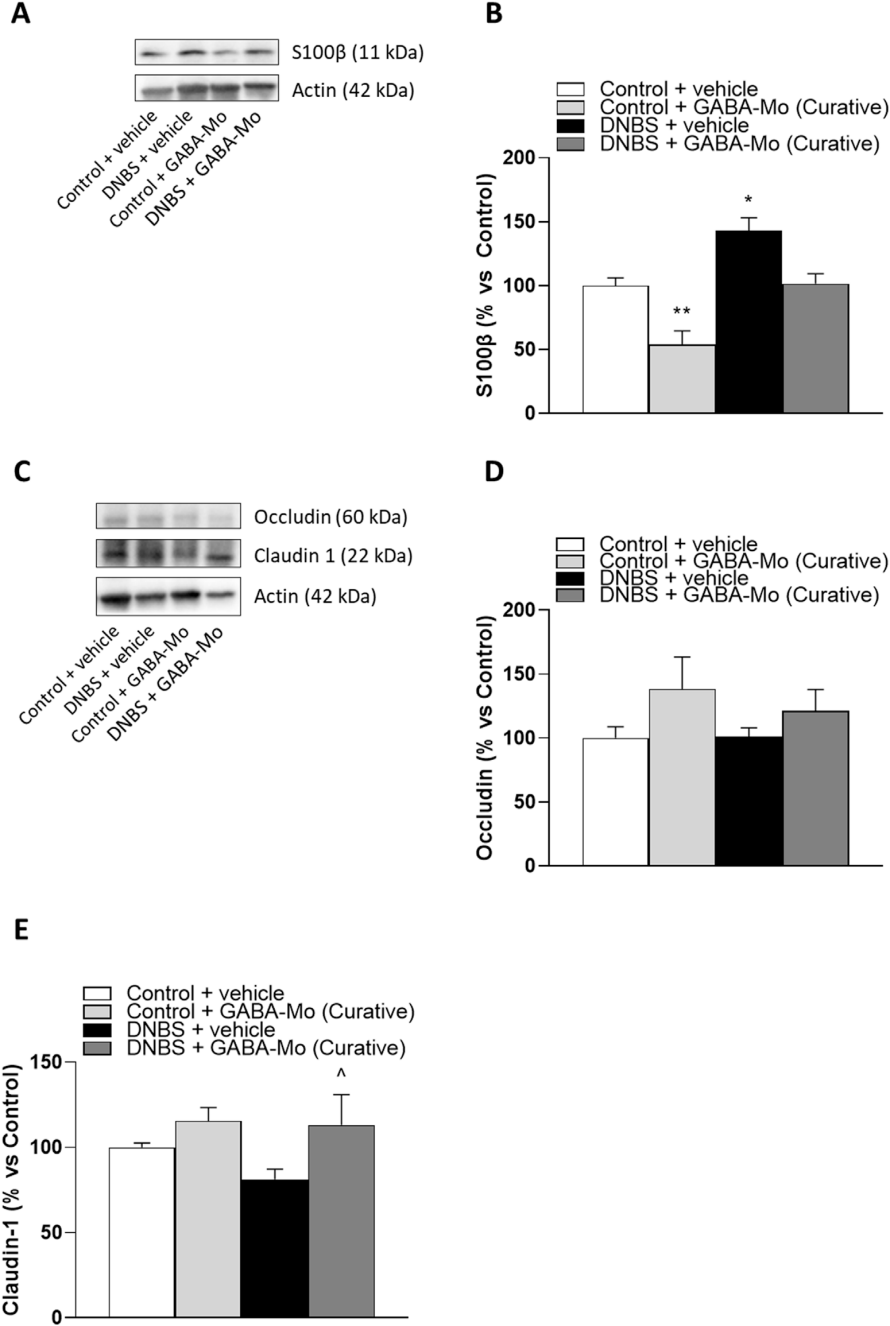
Curative protocol Representative blots **(A and C)** and densitometric analysis of the expression levels of S100β **(B)**, occludin **(D)** and claudin-1 **(E)** in control or DNBS rats treated with vehicle or GABA-Mo. Each column represents the mean ± SEM from eight animals. *P < 0.05, **P < 0.01 vs. Control + vehicle group. ^∧^ *P* < 0.05 vs. DNBS + vehicle group.

In the curative protocol, S100β expression levels were significantly raised in DNBS rats (Figures 13A, B), and GABA-Mo administration determined a significant reduction of this parameter in control animals. In addition, a reduction trend was observed in DNBS + GABA-Mo treatment group (Figures 13A, B). By contrast, occludin and claudin-1 expression levels were not significantly changed in DNBS-treated animals (Figures 13C–E), whereas supplementation with GABA-Mo significantly enhanced claudin-1 expression, as compared with DNBS group (Figures 13C, E).

### 3.8 Evaluation of plasma LBP

**FIGURE 14:**
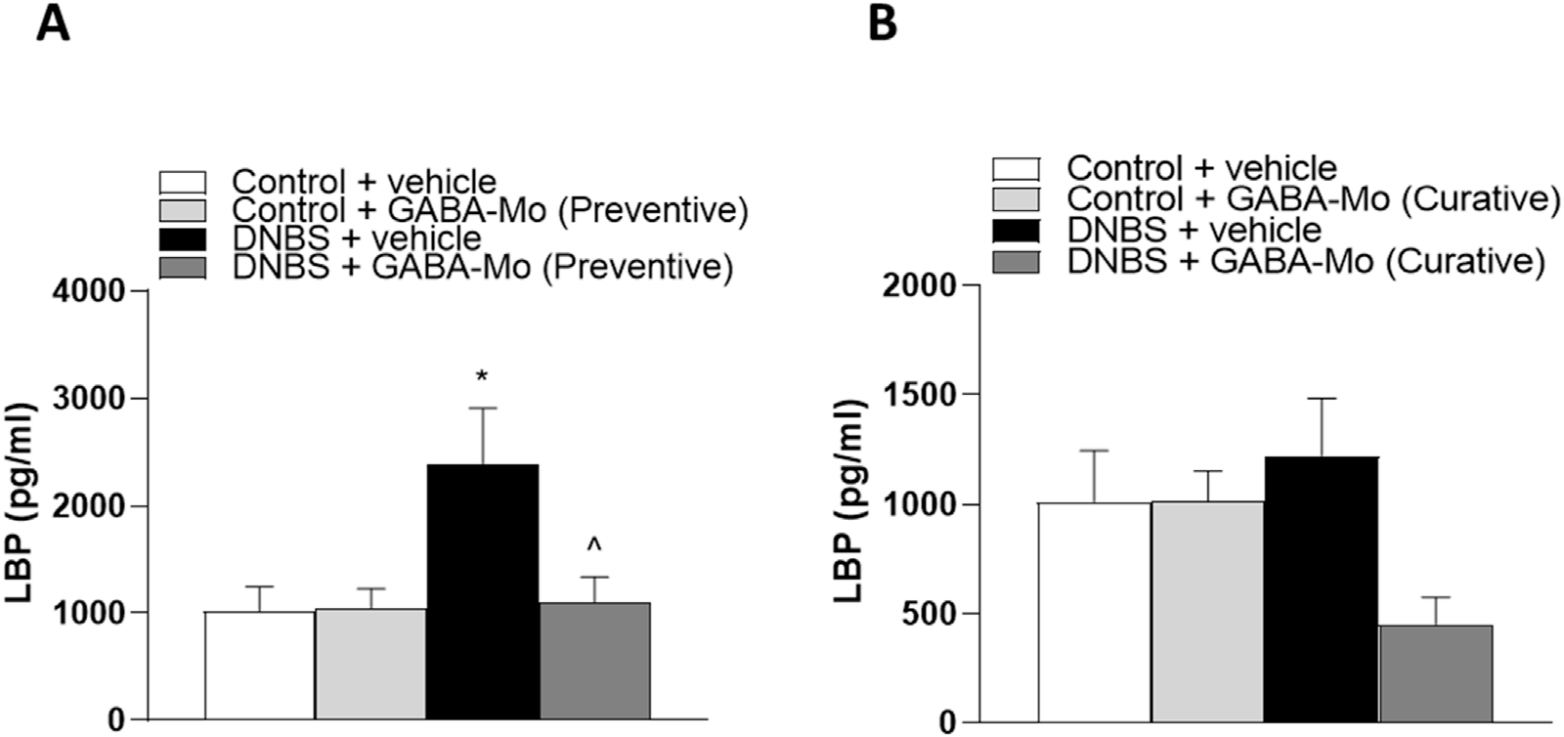
LBP levels in the preventive protocol of treatment **(A)** and curative protocol of treatment **(B)** in plasma from control or DNBS rats treated with vehicle or GABA-Mo. Each column represents the mean ± SEM from eight animals. *P < 0.05, **P < 0.01 vs. Control + vehicle group. ^∧^ *P* < 0.05 vs. DNBS + vehicle group.

In the preventive protocol, plasma LBP levels were significantly increased in DNBS rats, as compared with controls (Figure 14A). GABA-Mo intake significantly reduced this parameter (Figure 14A).

In the curative protocol, DNBS + GABA-Mo rats showed a trend in reduction of LBP levels, but the other treatment groups were not significantly altered (Figure 14B).

## 4 Discussion

Based on these preclinical results, it is possible to conclude that treatment with GABA-*Melissa officinalis*, especially in the curative protocol, can counteract the establishment and persistence of visceral hypersensitivity resulting from an inflammatory insult. The treatment was able to limit colon damage, tissue infiltration by polymorphonuclear cells and oxidative stress, as supported by decreasing actions against increased MPO and MDA levels, respectively. Although the tested drug had no effect on the levels of pro-inflammatory cytokines, GABA-Mo contributed to restore the intestinal epithelial barrier (IEB) integrity, confirmed by lower LBP levels and enhanced claudin-1 expression, and to reduce both enteric and spinal glial cell activation, phenomenon associated with pain chronicity. IBS-like symptoms, like abdominal pain and intestinal dysmotility, are reported in patients before the diagnosis of IBD or in the remission stage of inflammatory or infectious diseases. DNBS-induced visceral hypersensitivity persists after the resolution of the acute inflammatory phase, making this model suitable to investigate pain related to IBS conditions and therapies (Lucarini et al., 2020; Spiller and Major, 2016; Tsang et al., 2016; Dockerty et al., 2011). The main issues in the employment of classical analgesics for the management of chronic abdominal pain is the limited efficacy and the unavoidable side effects occurring in long-term therapies (Catanzaro et al., 2015). On the other hand, the identification of new effective and safe strategies is hindered by the complexity of this kind of pain which has a multifactorial nature, involving the persistence of low-grade inflammatory processes, alterations in the enteric barrier and immune response within the gut, as well as the sensitization of pain pathways in the central nervous system (Cervero, 2014; Carabotti et al., 2015; Camilleri and boeckxstaens, 2023). Hence the need to develop a multitarget therapy exerting complementary and synergistic beneficial effects on the gut-brain axis to treat IBS-like syndromes. The present work demonstrated that a product containing GABA, such as GABA-Mo, might have this potential. As mentioned, post-inflammatory visceral hypersensitivity is sustained, at least in part, by the sensitization of colonic nociceptors. As the major inhibitory neurotransmitter, GABA plays pivotal roles in the regulation of pain signalling by directly acting on different types of receptors (GABAA, GABAC and GABAB). The chloride-permeable ion channel receptors GABAA and GABAC mediate fast neurotransmission, while the metabotropic GABAB receptor mediates slow response. GABAB receptors are widely expressed in the gastrointestinal system, including enteric nervous system, where they are involved in the regulation of intestinal motility and visceral sensitivity (Hyland and Cryan, 2010). A recent preclinical study demonstrated diazepam capacity to decrease VMR in both DSS-treated and control mice by the modulation of peripheral GABAA receptor signalling (Loeza-Alcocer and Gold, 2022). GABAA receptor antagonists were reported to increase the colonic afferent excitability, suggesting that in the absence of tissue injury, there is sufficient extracellular GABA for GABAA receptor activation. Noteworthy, a decrease in the extracellular GABA occurs in the colon under inflammatory conditions (Aggarwal et al., 2017). GABAA receptors are widely expressed also in the central nervous system, where a loss of GABAA receptor inhibition can contribute to inflammatory hypersensitivity (Carlton et al., 1999; Anseloni and gold, 2008; Qian et al., 2023). Therefore, visceral pain might involve a decrease in GABA-mediated suppression of nociceptive signaling from the colon to central nervous system (Loeza-Alcocer and Gold, 2022). Within the gut lumen, a relevant amount of GABA is also produced by the commensal *Bifidobacterium dentium*, whose supplementation resulted able to modulate sensory neuron activity in a rat model of visceral hypersensitivity caused by fecal retention (Pokusaeva et al., 2017). Noteworthy, dysbiosis resulting in an impairment of bacterial metabolism and crosstalk with the intestinal epithelium emerges as a common trait of painful gastrointestinal diseases, both at preclinical and clinical level (Guo et al., 2019; Lucarini et al., 2022; Shaikh et al., 2023). Therefore, GABA supplementation might make up for both reduced level of specific *Bifidobacteria* and altered GABAergic system, under inflammatory conditions. Recently, Gold and Loeza-Alcocer (2024) demonstrated that increasing gastrointestinal levels of GABA may be useful for the treatment of colitis induced by DSS in mice. In the present work, we observed that GABA-based treatment is more effective in counteracting post-inflammatory visceral pain persistence (curative protocol) rather than in preventing its establishment during inflammatory conditions (preventive protocol), likely because neuroplasticity occurring in the peripheral and central nervous system after an inflammatory insult might make animals more responsive to the neurotransmitter supplementation. A maladaptive response of nervous systems common to several chronic painful conditions and contributing to post-inflammatory visceral pain persistence is the activation of glial cells at spinal cord level (in terms of either proliferation or functional changes; Lucarini et al., 2020). In the central nervous system, astrocytes undertake critical roles in pain pathophysiology, as through the secretion of transmitters, reactive astrocytes can influence primary afferent neuronal signalling or sensitize second-order neurons in the spinal cord (Pekny et al., 2016). In addition, astrocytes can alter pain perception through the nociceptive pathway by creating astrocytic networks capable of transducing signals for extended distances across and along the spinal cord, up to the brain (Hald, 2009; Lucarini et al., 2021). Noteworthy, spinal reactive astrogliosis has been associated with persistent visceral pain in DNBS-treated animals (Lucarini et al., 2020). The activation of glial cells at spinal cord level is stressed by the continuous overactivation of nociceptors which are responsible to alarm the brain of the ongoing pathological condition. The repeated administration of GABA-Mo in DNBS animals, by counteracting nociceptor transmission, might defuse the main mechanism of central sensitization of pain. Moreover, astrocytes are both GABAceptive and GABAergic cells, able to capture and internalize extracellular GABA, thus influencing both inhibitory and excitatory neurotransmission (Liu et al., 2022). Astrocyte activation can cause dysregulation of glutamate and GABA, leading to an imbalance of excitatory and inhibitory neuronal inputs, which in turn enhances pain signals (Li et al., 2019). In this context, the supplementation of GABA-Mo might restore a physiological condition. Another point to consider is that the transformation of astrocytes from normal to reactive phenotype involves a variety of intercellular and intracellular mechanisms, as signalling molecules released by neurons and microglia (Karimi-Abdolrezaee and Billakanti, 2012). Also, activation of microglia has been observed in several chronic pain models, including those reproducing visceral pain (Cao and Zhang, 2008; Lucarini et al., 2020). Preclinical evidence indicates that, during the development of chronic pain, microglia responses are typically early and transiently, contributing to the initiation of pain, while astrocyte activation occur later and last longer than microglia, thus becoming responsible for pain maintenance (Cao and Zhang, 2008; Raghavendra et al., 2003). This hypothesis is in line with the present findings, as preventive treatment with GABA-Mo mainly influenced microglia reactivity, while the curative intervention prevented astrocytes proliferation in the spinal cord of post-colitis animals. Anyway, though both microglia and astrocytes are GABA-responsive as well as GABA-ergic cells, further studies are needed to understand whether GABA-Mo directly or indirectly influence glia activity.

Besides the effects on CNS glia and microglia, recent evidence supports the involvement of GABA in the modulation of enteric glial cells activity both in physiological and pathological conditions. Moreover, it has been recognized a bidirectional role of GABA in communication among EGCs, neurons and immune system. For instance, GABA released by enteric glial cells take part to the regulation of intestinal motility by modulating neuronal circuitries activity (Seguella and Gulbransen, 2021). Under pathological conditions characterized by intestinal inflammation, enteric glia activation can contribute to enteric neuroplasticity and associated dysmotility and visceral pain (Morales-soto and Gulbransen, 2019), which have a main role in the development of typical symptoms observed in both inflammatory and functional bowel disorders (Seguella and Gulbransen, 2021). Furthermore, Deng et al. (2023) observed that GABA elicited significant anti-inflammatory effects in a mouse model of LPS-induced inflammation via activation of GABA receptors on enteric glia and subsequent decrease in cell activation. Taken together, these findings allow to hypothesize that GABAergic pathways activation could exert beneficial actions in different intestinal disorders, characterized by inflammation, dysmotility and pain through direct modulation of enteric glia activity. However, despite the above considerations, further research is needed to characterize the activity of GABA-Mo on enteric glial cells and related putative beneficial effects on pain and inflammation.

The formulation with *Melissa officinalis* in the preparation was conceived to support the beneficial effects of GABA, either by preventing its metabolism *in vivo* or by a direct modulation of nociception (Awad et al., 2007; Giuginski et al., 2009; Dolatabadi et al., 2018; Chindo et al., 2024). Melissa has been reported to attenuate acute and chronic induced nociception via ATP-sensitive potassium channel (KATP channel), opioidergic and serotonergic pathways (Chindo et al., 2024). Moreover, the administration of an aqueous extract of Melissa was demonstrated to effectively inhibit GABA-transaminase activity (Awad et al., 2007), a mechanism able to prolong the half-life of GABA and its downstream effects on the different cellular targets. As the single constituents (Ngo and Vo, 2019; Zam et al., 2022; Abd Alah et al., 2022), GABA-Mo exerted protective effects against both inflammation and oxidative stress caused by DNBS injection in rats. In recent years, growing evidence has pointed to a causal relationship between chronic bowel inflammation, visceral pain, and IEB disruption. Actually, the inflammatory condition in the digestive tract, in addition to contributing to nociceptor sensitization, brings to the weakening of the IEB, contributing to a condition known as “leaky gut,” which, in turn, promotes the worsening of the inflammatory processes (Michielan and D'Incà, 2015; Vindigni et al., 2016). Beyond neuromodulation, GABA can protect from inflammation by modulating cytokines production (Gros et al., 2021). Also in our study, we observed that the GABA-Mo supplementation was able to reduce intestinal inflammation and oxidative stress in DNBS-induced colitis, and was able to partially restore IEB integrity, as evidenced by decreased LBP levels and increased claudin-1 expression. In line with our results, products containing GABA have been proven to strengthen IEB and to modulate immune-inflammatory response (Sokovic Bajic et al., 2019; Cataldo et al., 2020). Although the treatment with GABA-Mo did not significantly alter normal visceral sensitivity neither the investigated histological and molecular parameters, the slightly increase in the AWR and VMR values observed in the GABA-Mo treated controls with respect of vehicle-treated controls is an aspect to further monitor in the view of long-lasting therapies.

In conclusion GABA-Mo, considering its broad-spectrum efficacy, represents a suitable approach to promote the restoration of gut health and to improve the therapy of chronic abdominal pain, especially in the remission phase of intestinal inflammation.

## Data Availability

The raw data supporting the conclusions of this article will be made available by the authors, without undue reservation.
